# Alterations of fecal short-chain fatty acids solely in the course of multiple sclerosis: rethinking the gut–brain axis in the early stages of MS

**DOI:** 10.1177/17562864251396028

**Published:** 2025-12-12

**Authors:** Jakob Stögbauer, Niklas Kämpfer, Anouck Becker-Dorison, Andreas Schwiertz, Sergiu Groppa, Marcus M. Unger, Mathias Fousse

**Affiliations:** Department of Neurology, Saarland University, Campus Homburg, Building 90, Kirrberger Straße, Homburg 66421, Germany; Department of Neurology, Saarland University Medical Center, Homburg, Germany; Department of Neurology, Saarland University Medical Center, Homburg, Germany; Department of Neurology, Klinikum Saarbrücken, Saarbrücken, Germany; MVZ Institute of Microecology, Herborn, Germany; Department of Neurology, Saarland University Medical Center, Homburg, Germany; Department of Neurology, SHG Kliniken Sonnenberg, Saarbruecken, Germany; Department of Neurology, Saarland University Medical Center, Homburg, Germany

**Keywords:** inflammation, microbiome, microbiota, multiple sclerosis, short-chain fatty acids

## Abstract

**Background:**

The role of gut microbiota in multiple sclerosis (MS) has become increasingly important, intestinal dysbiosis with reduced production of short-chain fatty acids (SCFA) being the prevailing paradigm. However, the direction of causality, that is, whether intestinal changes are cause or consequence of chronic central nervous system inflammation, remains to be elucidated. Previous studies have focused on long-term MS patients. Alteration in fecal SCFA concentrations in early MS, particularly during relapses, remains to be extensively studied.

**Objectives:**

To compare fecal SCFA concentrations in patients with a first diagnosis of MS with those in patients with long-term MS and in healthy controls (HCs).

**Design:**

Prospective cohort study.

**Methods:**

The prospective case–control study was conducted on relapsing-remitting MS (RRMS) patients at the time of first, acute relapse without ongoing immunotherapy (Early-RRMS). Clinical and demographic parameters, as well as fecal SCFA concentrations (measured by gas chromatography) were collected. The parameters were compared with those of matched RRMS patients under different, long-term immunotherapy (Late-RRMS) and HCs.

**Results:**

SCFA concentrations of propionate, butyrate, isobutyrate, valerate, and isovalerate were not significantly different between the early-RRMS cohort and HCs, but were lower in the late-RRMS cohort.

**Conclusion:**

The findings indicate that reduction in SCFA levels is exclusively observed in patients with RRMS during the further course of the disease and not at the onset. Decrease in SCFA concentration may be rather consequence or related to neurodegeneration than linked to the first demyelinating event. Further investigation related to disease trajectories of immunomodulatory or neuroprotective treatments are required.

## Introduction

Multiple sclerosis (MS) is a chronic inflammatory disease of the central nervous system (CNS) that is the most common nontraumatic cause of disability in young people.^1^ In MS, sustained inflammation can result in progressive central nervous demyelination and subsequent axonal damage.^2,3^ Despite the numerous research studies conducted on the subject, the etiology of the disease has not yet been conclusively clarified; it is generally assumed that genetic and environmental factors interact.^4–7^ In relation to the latter, in addition to infections (e.g., reactivation of Epstein–Barr virus) and disorders of vitamin D balance, changes in the brain–gut axis with intestinal dysbiosis are also being discussed. These changes have become a center of interest in recent years.^4,8–10^

Individuals diagnosed with MS seem to demonstrate an alteration in their gut microbiota. Specifically, a reduced number of bacteria that produce short-chain fatty acids (SCFA) was found compared to healthy controls (HCs).^10^ Consequently, alterations in the concentration and composition of the SCFA in patients with MS occur. These changes can affect a wide range of fatty acids: a reduction in butyrate (BA) is observed, an organic compound which has anti-inflammatory properties and plays an essential role in intestinal barrier function.^11^

A reduced intestinal concentration of SCFAs is generally considered to promote an inflammatory environment.^12^ A plethora of animal experiments have demonstrated that the pathogenesis and progression of experimental autoimmune encephalomyelitis (EAE), the accepted animal model of MS, can be influenced positively by SCFAs.^13^ In preclinical studies, supplementation of SCFAs (e.g., BA, propionate (PA), valerate) was found to be capable of suppressing demyelination and increasing remyelination.^14,15^ The ingestion of PA has been demonstrated to engender positive clinical effects in patients diagnosed with MS.^15^ However, in addition to the protective effects previously documented, pro-inflammatory effects were also demonstrated in EAE experiments.^16^

It is important to note that SCFAs are essential for the brain–gut axis due to their ability to cross the blood–brain barrier (BBB)^17^ and their interaction with immune cells (especially T lymphocytes and cytokines^18^), and that they therefore seem to have an impact on the course of MS.

However, at present, no clear cause–effect relationship between intestinal and CNS inflammation has been identified. The direction of causality, that is, whether intestinal changes are cause or consequence of chronic CNS inflammation, remains to be elucidated. Additionally, it should be emphasized that the effect of immunomodulatory therapies on the microbiota has so far been insufficiently investigated. Some data suggest that treatment with interferon beta can lead to a kind of “restorement” of the gut microbiota and eubiosis.^19^ The sample sizes are small and the effect on SCFAs remains uncharted.

Another inflammation marker established in the context of inflammatory bowel diseases is fecal calprotectin, which has also been shown to be elevated in various other diseases as a marker of gut immune system activation and intestinal inflammation.^20,21^ Consequently, conducting an investigation into active MS patients appears to be a logical next step.

SCFA studies in patients with MS are not yet widespread, despite the existence of encouraging preclinical data. The majority of previous studies^10^ included patients who, although not always treated, had been suffering from the disease for many years. Furthermore, the analysis primarily focused on plasma concentrations rather than fecal concentrations. Research is particularly lacking in relation to studies on patients in relapse or at the time of initial diagnosis, as well as case–control comparisons between patients in the early or late stages of the disease. The pathophysiological intestinal processes in patients with a first demyelinating event are entirely unknown.

The objective of this study is to evaluate alterations in SCFA levels during the initial phases of relapsing-remitting MS (RRMS) in comparison to individuals with known RRMS who are undergoing immunotherapy and to HCs. For this purpose, patients with the first manifestation of MS are included before the initiation of immunosuppressive therapy. This methodology has not been employed to date. The findings should assist in clarifying the question of when reduced excretion of SCFAs in MS occurs, and which associated pathophysiological conclusions can be drawn from this.

## Materials and methods

### Patients and data collection

Patients with an initial diagnosis of active RRMS (Early-RRMS) were assessed at the Department of Neurology of Saarland University Medical Center, Germany, between January 2024 and March 2025. All patients were included during their first relapse, prior to the initiation of steroid pulse therapy and within 12 h after administration. The diagnosis of RRMS was made using the revised McDonald criteria of 2017.^22^ Patients with a known disease of acute or chronic intestinal inflammation, a coexistent infection within the past 4 weeks and intake of antibiotics during the past 8 weeks were excluded. Following their inclusion in the study, patients were provided with a fecal sampling kit and comprehensive instructions on its utilization. The determination of fecal SCFAs and calprotectin concentrations was conducted in accordance with the established methodology of gas chromatography, as previously described.^23^

The collection of biographical and anamnestic information was derived from the patient file. The clinical examination was objectified using the Expanded Disability Status Scale (EDSS^24^). Furthermore, a comprehensive neuropsychological evaluation was conducted, encompassing the Beck Depression Inventory (BDI^25^), the Modified Fatigue Impact Scale (MFIS^26^), the Mini Mental State Examination (MMSE^27^), and a range of laboratory tests, including C reactive protein (CRP), leucocyte count, neutrophil and lymphocyte percentage, and vitamin D serum levels. The control groups comprised HCs without previous neurological or gastrointestinal conditions and patients with known active RRMS who were undergoing long-term immunotherapy (Late-RRMS). The subjects of the late-RRMS group were age- and sex-matched, the HC (people without known neurological or intestinal diseases) age-matched from a previous study conducted by this research group.^28^

All patients provided written consent for participation in the study. Study protocol was approved by the local ethics committee on November 2, 2021 (Ethikkommission der Ärztekammer des Saarlandes, vote No. 262/21).

### Statistical analysis

Statistical analysis was performed using SPSS statistics (IBM, version 29.0.2.0, Armonk, New York, USA). Initially, the data were subjected to Shapiro–Wilk test in order to ascertain the existence of a normal distribution. The descriptive statistics are described using mean and standard deviation or median and range. Spearman’s correlation coefficients were utilized to analyze correlations between metric variables. The Mann–Whitney *U* and Kruskal–Wallis tests were used to compare unpaired nonparametric data. Chi-square tests were used for analyzation of categorical data. Correlations were considered significant if *p*-value was <0.05 (with a statistical power of 80%).

## Results

### Patient characteristics

From January 2024 to March 2025, 22 patients (16 of whom were female) with newly diagnosed RRMS (early-RRMS) were included in this case–control, prospective study during their first relapse prior to initiation of steroid therapy. Comparisons were made with age-matched HC (*n* = 22) and age- and sex-matched patients with preexisting MS undergoing long-term disease-modifying treatment (late-RRMS, *n* = 22). Mean disease duration in the late-RRMS group was 11 years. The mean age of the patients was approximately 40 years, and no significant increase in CRP levels as an indicator of inflammation, was observed in any of the groups at the time of testing. History of smoking, vitamin D level, and leucocyte count was known for the early-RRMS cohort only. The median vitamin D level was found to be marginally low in the early-RRMS group. A neuropsychological examination was conducted on all 66 patients. Statistically significant differences were absent in relation to the presence of cognitive dysfunction or depression, as measured by the MMSE and BDI, respectively. The late-RRMS cohort demonstrated a statistically significant increase in MFIS scores, indicative of a greater degree of impairment due to fatigue.

The two RRMS groups did not differ significantly in terms of clinical impairment as measured by the EDSS score.

The patient characteristics are displayed in Table 1.

**Table 1.** Patient characteristics.

Characteristics	Early-RRMS	Late-RRMS	HC	*p*
*n*	22	22	22	
Sex category female *n* (%)	16 (72.7)	16 (72.7)	10 (45.5)	0.095
Age *x* ± *s*	39.86 ± 12	38.2 ± 8.3	39.1 ± 11.6	0.886
Disease modifying treatment, *n* (%)	0 (0)	21 (95.5)	0 (0)	
Interferon beta		2 (9.1)		
Glatiramer acetate		2 (9.1)		
Dimethyl fumarate		5 (22.7)		
S1P modulation		2 (9.1)		
Natalizumab		10 (45.5)		
Disease duration Years *x* ± *s*	0	10.9 ± 8.6		
Nicotine yes/no *n* (%)	5 (22.7)/17 (77.3)			
CRP median (range) (mg/dl)	1.1 (0.6–23)	1.45 (1–9.6)	1.4 (1–14)	0.148
Leucocytes/µl median (range)	7030 (5455–8701)			
% neutrophiles median (range)	62.9 (39–83.5)			
% lymphocytes median (range)	30.3 (16.6–92)			
Serum vitamin D level median (range) (ng/ml)	24.8 (12.3–92)			
Neuropsychological testing				
MMSE median (range)	29.5 (27–30)	29 (23–30)	29 (26–30)	0.395
MFIS median (range)	35.5 (2–65)	49 (2–118)	24.5 (0–93)	0.01
BDI median (range)	9 (0–28)	9.5 (1–33)	6 (0–21)	0.255
EDSS median (range)	2 (1–4.5)	2.25 (0–7)		0.844

Normal ranges for laboratory findings: CRP <5 mg/dl; leucocytes 3900–10,200/µl; % neutrophiles 42%–77%; % lymphocytes 20%–44%; serum vitamin D level 30–100 ng/ml. Ranges for test items: MMSE 0–30, MFIS 0–84; BDI 0–63.

BDI, Beck’s Depression Inventory; CRP, C reactive protein; EDSS, Expanded Disability Status Scale; HC, healthy controls; MFIS, Modified Fatigue Impact Scale; MMSE, Mini Mental State Examination; RRMS, relapsing-remitting Multiple Sclerosis.

### Fecal SCFA concentrations

All patients included in the study provided a stool sample, which was then analyzed for concentrations of SCFAs according to the procedure previously described.^23^ Statistically significant differences were not observed in the fecal concentrations of acetate (*p* = 0.577), propionate (*p* = 0.561), butyrate (*p* = 0.354), isobutyrate (*p* = 0.969), valerate (*p* = 0.25), and isovalerate (*p* = 0.206) between the early-RRMS cohort and the HC.

The comparison of early-RRMS patients with late-RRMS patients revealed that statistically significant higher concentrations of SCFAs were consistently observed in the initial diagnosis group. This applied to all SCFAs analyzed with the exception of acetate. In patients with late RRMS, there were no differences in SCFA concentrations between the various treatment groups. The same was true when the therapeutic agents were categorized as either induction treatment (interferon beta, glatiramer acetate, or dimethyl fumarate) or escalation treatment (S1P modulation or natalizumab; *p* > 0.05 in each case).

**Figure 1.**
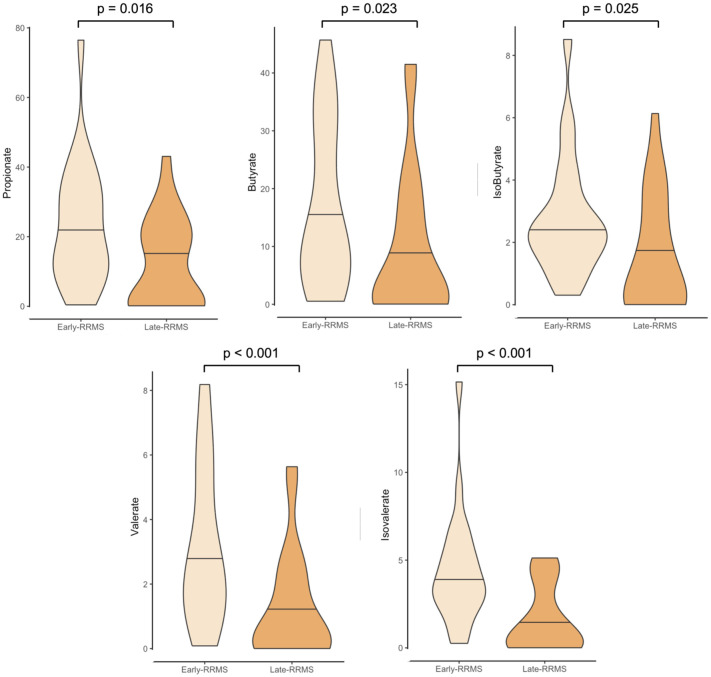
Violin plots show fecal SCFA concentrations in early-RRMS patients and late-RRMS patients. SCFA concentrations are described in mmol/g. Outliers are not depicted for better visualization. *p*-Values calculated by Mann–Whitney *U* test. RRMS, relapsing-remitting multiple sclerosis; SCFA, short-chain fatty acid.

The group comparisons are demonstrated in Table 2 and further illustrated in Figure 1 to facilitate comprehension.

**Table 2.** Fecal SCFA concentrations in early-RRMS patients and late-RRMS patients.

Type of SCFA	Late-RRMS	Early-RRMS	*p*
Acetate median (range) (mmol/g)	32.3 (0.3–160.2)	58.3 (0.7–193.1)	0.102
Propionate median (range) (mmol/g)	5.8 (0.1–43.1)	17.6 (0.2–87.8)	**0.023**
Butyrate median (range) (mmol/g)	1.8 (0.04–41.5)	13.1 (0.09–52.5)	**0.016**
Isobutyrate median (range) (mmol/g)	0.99 (0.004–6.1)	2.76 (0.023–11.35)	**0.025**
Valerate median (range) (mmol/g)	0.57 (0.002–5.6)	1.47 (0.024–19.8)	**<0.001**
Isovalerate median (range) (mmol/g)	0.86 (0.007–5.1)	2.48 (0.037–17.03)	**<0.001**

RRMS, relapsing-remitting multiple sclerosis; SCFA, short-chain fatty acids.

Significant correlations are marked bold.

No statistically significant correlations were identified between fecal SCFA concentrations and EDSS, vitamin D levels, leucocyte, neutrophil and lymphocyte counts, gender and history of smoking. The same was true for the relationship to cognition (measured by MMSE), depression (measured by BDI), and fatigue (measured by MFIS).

### Fecal calprotectin concentration

Patients in the early-RRMS group exhibited marginally elevated concentrations of fecal calprotectin in comparison to the control populations (HC and Late-RRMS). However, no statistically significant differences were observed (Table 3).

**Table 3.** Fecal calprotectin concentrations in early-RRMS patients, late-RRMS patients, and HC.

Subject	Early-RRMS	Late-RRMS	HC	*p*
Calprotectin median (range) (µg/g)	19 (19–730)	19 (19–82)	19 (19–328)	0.385

HC, healthy controls; RRMS, relapsing-remitting multiple sclerosis.

The calprotectin concentrations were found to be independent of the EDSS (*p* = 0.697), scores of MMSE (*p* = 0.753), BDI (*p* = 0.803), and MFIS (*p* = 0.861), vitamin D level (*p* = 0.729), leucocyte count (*p* = 0.245), neutrophil count (*p* = 0.562), lymphocyte count (*p* = 0.337), and history of smoking (*p* = 0.617).

## Discussion

In the present prospective cohort study, fecal samples were collected from 44 patients with RRMS and 22 HC. In patients with an initial diagnosis of RRMS, SCFA and calprotectin concentrations were analyzed at the time of first relapse and subsequently compared to the results of HC and patients with known RRMS undergoing long-term immunotherapy. In contrast to the findings of preceding studies, it was demonstrated that the fecal SCFA concentration of newly diagnosed MS patients did not exhibit a discrepancy in comparison to age-matched controls.^8–10^ However, the early-RRMS cohort demonstrated significantly higher fecal SCFA concentrations in comparison to the late-RRMS cohort. The results merit particular consideration as we have conducted a head-to-head comparison between MS patients at varying stages of the disease (early to late, relapse to steady state). Previous SCFA studies have generally focused on long-term, often treated patients without a current relapse event.^9,10^ In addition, an analysis was conducted on fecal concentrations, which may have yielded more realistic values than those obtained from plasma concentrations.

Reduced fecal SCFAs are widely accepted as a contributing factor to inflammatory responses.^12^ Given that these substances have been shown to permeate the BBB, it is widely accepted that they can potentially impact a range of autoimmune CNS diseases. Concentrations of SCFAs in MS patients have been shown to be low in previous studies,^10^ which has led some authors to hypothesize that intestinal inflammation may be a contributing factor to secondary CNS inflammation.^29^ Conversely, there is also evidence to suggest that CNS injuries can induce increased intestinal permeability, which can in turn lead to dysbiosis.^30^ The hypothesis postulated here is that intestinal activation of CD4 positive T cells occurs, which then initiates autoimmunity in the CNS.^31^ However, the causal relationship between these factors remains to be elucidated, and the bidirectional nature of the gut–brain axis is undoubtedly a complex mechanism.

The results of our study allow us to hypothesize that low intestinal SCFA concentrations may only occur during the course of the disease and could therefore be a consequence of chronic CNS inflammation itself. Nevertheless, at the onset of the disease, the SCFAs of patients with MS seem to be not different from those of healthy individuals.

A further influencing factor is the immunomodulatory therapies of MS patients, the effect of which on the gut microbiota has not yet been the subject of sufficient investigation, and only for individual drugs.^19^ It is conceivable that these may also result in alterations to the microbiome, which, in subsequent instances, may lead to changes in the SCFAs. The hypothesis that the altered SCFA concentration could be drug-induced and not due to MS inflammation may be supported by the fact that the EDSS is the same in both MS groups despite the different lengths of disease progression. It is evident that systematic studies for individual treatment groups are required in this context.

Another possible explanation for SCFA alterations in the later stages of MS is their connection to the onset of neurodegeneration rather than inflammation. This is consistent with similar alterations being observed in classic neurodegenerative diseases such as Alzheimer’s^32^ and Parkinson’s.^23,33^

The fecal concentration of calprotectin has been established as an intestinal inflammation marker in the context of IBD. In MS patients undergoing long-term immunotherapy, no increase in fecal calprotectin has been demonstrated to date,^28^ data concerning untreated patients or patients during relapses are lacking. Nevertheless, an increase in fecal calprotectin has been evidenced in EAE experiments.^34^ Based on these preclinical findings, it was hypothesized that calprotectin would be elevated in untreated MS patients during relapses. Conversely, the present study failed to identify any significant differences between the observed groups. However, a descriptive increase in the early-RRMS patients was identified in comparison to the late-RRMS group. This finding lends support to the hypothesis that intestinal involvement plays a role in the context of systemic inflammation in MS patients. A significant proportion of the MS patients treated had natalizumab as a therapeutic agent, which is also effective in the treatment of chronic inflammatory bowel disease.^35^ However, changes in SCFA have also been described in relation to other immunotherapeutic agents. It is imperative that future studies with larger numbers of patients be conducted in order to ascertain the relevance of this effect.

As mentioned above, it is thought that many different environmental factors influence the pathogenesis of MS, and some of these may also impact the microbiome.^36^ However, in the case of vitamin D at least, we failed to demonstrate a statistically significant correlation between serum levels and SCFA concentrations. Future studies could systematically analyze correlations between SCFAs in treatment-naïve MS patients and the different risk factors.

It is important to note that, in the present study, no direct correlation was identified between SCFA or calprotectin concentrations and clinical parameters (as measured by EDSS, BDI, MMSE, and MFIS). In patients diagnosed with myalgic encephalomyelitis/chronic fatigue syndrome, previous studies have demonstrated a correlation between the severity of fatigue and reduced intestinal butyrate concentrations. We were not able to demonstrate this phenomenon in patients suffering from MS.^37^ Further research is required in order to elucidate the relationship between microbiome changes and the clinical presentation of MS patients.

### Limitations

The primary constraint of this study is the limited number of patients who were included in the analysis. Despite this limitation, we were able to identify significant and consistent differences between the various patient groups. The findings of this study require further validation through expanded research endeavors. It is also important to mention the heterogeneity of the two RRMS cohorts. While the early RRMS patients were in the acute phase of the disease, the late RRMS patients had been ill for many years and also received immunomodulatory therapy, which could also have an impact on the intestinal microbiota. Additionally, time since the last relapse for the late-RRMS patients was not available. Further studies are needed in this area.

MMSE was chosen to assess cognitive dysfunction. However, this may not be the ideal choice as it was initially designed for patients with dementia rather than MS. Future studies should include other cognitive tests that are specific to MS.

A further limitation is the absence of data regarding dietary habits, which have been demonstrated to influence the composition of the gut microbiota. In the course of the present study, other influencing factors were excluded, including systemic inflammation due to infection, medication intake, and intestinal comorbidities.

## Conclusion

Our findings indicate that reduction in SCFA levels is exclusively observed in patients with RRMS during the further course of the disease, rather than at the onset. It can be hypothesized that decrease in SCFA concentration is rather consequence than cause of chronic CNS inflammation. Moreover, the present study discovered evidence indicative of an elevated fecal calprotectin concentration during relapses. Further investigation of these connections is required, particularly with the use of EAE models.

## Appendix

### Abbreviations

BBB blood-brain barrier

BDI Beck Depression Inventory

CNS central nervous system

EAE experimental autoimmune encephalomyelitis

EDSS Expanded Disability Status Scale

HC healthy controls

MFIS Modified Fatigue Impact Scale

MMSE Mini Mental State Examination

MS multiple sclerosis

RRMS relapsing-remitting multiple sclerosis

SCFA short-chain fatty acid

*x* mean

## Declarations

**Ethics approval and consent to participate:** All procedures performed in this study involving human participants were in accordance with the ethical standards of the institutional research committee and with the 1964 Helsinki Declaration and its later amendments or comparable ethical standards. The study was approved by the local ethics committee (Ethics Committee of the Saarland Medical Association, identification number 262/21). All patients gave their written consent to participate in the present study.

**Consent for publication:** All patients gave their written consent for publication of their data.

**Availability of data and materials:** All figures have associated raw data. The data that supports the findings of this study is available from the corresponding author upon reasonable request.
